# Intraspecific variations in life history traits of two pecky rice bug species from Japan: Mapping emergence dates and number of annual generations

**DOI:** 10.1002/ece3.8329

**Published:** 2021-11-12

**Authors:** Kazuhisa Yamasaki, Ken Tabuchi, Akihiko Takahashi, Takeshi Osawa, Akira Yoshioka, Yasushi Ishigooka, Shigeto Sudo, Mayura B. Takada

**Affiliations:** ^1^ Institute for Sustainable Agro‐Ecosystem Services Graduate School of Agricultural and Life Sciences The University of Tokyo Tokyo Japan; ^2^ Faculty of Agriculture Tokyo University of Agriculture and Technology Tokyo Japan; ^3^ Division of Crop Rotation Research for Lowland Farming Tohoku Agricultural Research Center NARO Iwate Japan; ^4^ Hokuriku Research Station Central Region Agricultural Research Center NARO Niigata Japan; ^5^ Graduate School of Urban Environmental Sciences Tokyo Metropolitan University Tokyo Japan; ^6^ Fukushima Regional Collaborative Research Center National Institute for Environmental Studies Fukushima Japan; ^7^ Hokkaido Agricultural Research Center NARO Hokkaido Japan; ^8^ Institute for Agro‐Environmental Sciences NARO Ibaraki Japan; ^9^ Faculty of Science and Engineering Chuo University Tokyo Japan

**Keywords:** development rate, effective accumulated temperature, geographic variation, local adaptation, risk map

## Abstract

The mirid bugs *Stenotus rubrovittatus* and *Trigonotylus caelestialium*, which cause pecky rice, have become a threat to rice cultivation in Asia. Damage caused by these pests has rapidly become frequent since around 2000 in Japan. Their expansion pattern is not simple, and predicting their future spread remains challenging. Some insects with wide ranges have locally adapted variations in life‐history traits. We performed laboratory rearing experiments to assess the geographical scale of intraspecific variations in life‐history traits of *S. rubrovittatus* and *T*. *caelestialium*. The experiments were aimed at increasing the accuracy of occurrence estimates and the number of generations per year. These results were compared with previous research, and differences in development rates were observed between populations of different latitudes, but not of the same latitude. Finally, plotting the timing of adult emergence and the potential number of generations per year on maps with a 5‐km grid revealed that they differed greatly locally at the same latitude. These maps can be used for developing more efficient methods of managing mirid bugs in integrated pest management.

## Graphical abstract

Our laboratory rearing experiments revealed no difference in the relationship between temperature and the rate of development in each developmental stage of the two mirid bug species from the same latitude in northern Japan. On the other hand, comparison of data on the development rates in Tohoku region and those of previous studies in other areas revealed that the relationship between temperature and the rate of development showed geographical variations between populations of both species at different latitudes. We mapped the timing of adult emergence of the first generation and the potential number of generations per year using the daily mean temperature and our data on the two species’ development in an effective accumulated temperatures model.

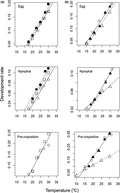

## 1 INTRODUCTION

Insects, being poikilotherms, are subjected to strong selection pressure to adapt their life history traits to habitat temperature (Chown & Terblanche, 2007; Colinet et al., 2015). Knowledge of the influence of temperature on insects is therefore crucial to understand changes in seasonal patterns of phenology (Kojima et al., 2020; Powell & Logan, 2005), distribution (Régnière et al., 2012), and population dynamics (Gray, 2008; Kingsolver, 1989). The relationships between the rate of development of insects and temperature have been studied in many insect species to test various hypotheses of their patterns of adaptation to temperature (Kipyatkov & Lopatina, 2010). Previous studies have focused mainly on comparison of temperature‐dependent development among species or genus, and there are few studies of geographical intraspecific variations in development. The rate of development of less mobile insect species with wide distributions can be optimized to the local climate (Higaki & Ando, 2002), resulting in geographical intraspecific variations in the rate of development.

Temperature‐dependent development of insect pests allows prediction of dates of emergence, the timing of insecticide applications (Tang & Cheke, 2008), the risk of outbreaks, and the number of generations per year (Moore & Remais, 2014). Some studies have used degree‐day models that assume a linear relationship between the rate of development and temperature (Easterbrook et al., 2003; Taylor, 1981) obtained from one or a few populations of a species. However, unless the extent of geographic variation in temperature‐dependent development among populations and the spatial scale at which the variation is determined are known, it remains difficult to explain and predict population dynamics of a species accurately. Understanding such variations is essential for establishing effective, locally adapted pest management strategies, such as predicting and mapping the timing of dates of emergence and the number of generations per year.

Pecky rice bugs are among the most serious economic insect pests of rice (*Oryza sativa* L.) in Asia and the USA (Ane & Hussain, 2016; Pathak & Khan, 1994; Tindall et al., 2005). By feeding on rice grains, they leave black spots. Rice showing this type of damage is called pecky rice (Kiritani, 2007; Tindall et al., 2005). In Japan, two mirid bug species—*Stenotus rubrovittatus* (Matsumura) and *Trigonotylus caelestialium* (Kirkaldy) (Hemiptera: Miridae)—have rapidly increased their distribution ranges and population densities since around 2000 (Ohtomo, 2013; Pathak & Khan, 1994; Tabuchi et al., 2015). Damage caused by *S. rubrovittatus* was first reported in the 1980s in one prefecture in northern Japan (Miyagi Prefecture) and in one prefecture in western Japan (Hiroshima Prefecture), followed by an increase in damage in surrounding prefectures, and is now reported in most of Japan (Kobayashi et al., 2011; Watanabe & Higuchi, 2006). In the 1990s, *T*. *caelestialium* populations rose sharply in one prefecture in the central region (Niigata Prefecture), followed by an increase in numbers and damage in northern Japan (Watanabe & Higuchi, 2006). Even a low incidence of pecky rice grains leads to severe economic losses to Japanese farmers under the present national regulation system for rice quality, which depends exclusively on appearance of grains (Tabuchi et al., 2015; Watanabe & Higuchi, 2006). These two mirid bug species are considered to have spread because of global warming (Kiritani, 2007; Osawa et al., 2018b) and an increase in their source habitats such as fallow and meadow fields throughout Japan (Kiritani, 2007; Osawa et al., 2018a). However, only higher temperatures and more available habitats cannot systematically explain the difference in expansion patterns among prefectures, i.e., the initial multiple outbreaks of each species and the subsequent expansion process (Tabuchi et al., 2015). Therefore, it is important to understand the complex expansion pattern of these bugs to predict their future spread. One of the keys of the understanding may be a regional adaptation to spatial variations in the thermal environment.

Here, we investigated the extent of the geographical variations in life history traits of *S. rubrovittatus* and *T*. *caelestialium*, whose distribution ranges and damage have rapidly expanded throughout Japan in the last two decades. One of the reasons why their distribution expansion pattern differs among prefectures is likely to be population differences in adaptation to temperature. No studies have explicitly tested the geographical variation of temperature adaptation of the two species. Clarifying the spatial patterns of such geographic variation may reveal the mechanisms of their complex expansion patterns and thus contribute to the establishment of effective, locally adapted pest management strategies in integrated pest management. We compared their life history traits among populations at different spatial scales. We hypothesized that the traits differ between populations widely separated (1) at the same latitude and (2) at different latitudes. To test the first hypothesis, we examined the development times and body size of two geographically separated populations at similar latitudes in the Tohoku region, northern Japan, by rearing them in the laboratory. These two populations are separated by the Ou Mountains, with peaks of up to 2000 m in elevation. We examined the rates of development of the egg, nymph, and preoviposition stages under five constant rearing temperatures. To test the second hypothesis, we compared the rates of development in the Tohoku region revealed by our experiments and those of previous studies in other areas. We also tested whether the traits differ between sympatric populations of the two species in the Tohoku region. Finally, we mapped the timing of adult emergence of the first generation and the potential number of generations per year, considering the spatial extent of the geographic variation in temperature‐dependent development among populations of both species.

## 2 MATERIALS AND METHODS

### 2.1 Study species

*Stenotus rubrovittatus* and *T*. *caelestialium* are native Japanese mirid bugs widely distributed throughout Japan (Sato & Yasunaga, 1999; Watanabe & Higuchi, 2006). The adults are ~5 mm in length. Because *S. rubrovittatus* usually feeds on grasses and sedges, it inhabits fallows and meadows around paddy fields (Shimada & Sugiura, 2021; Takada et al., 2012), where it reproduces and overwinters (Yoshioka et al., 2011). The bugs draw sap from and oviposit in the ears of host plants. The species has a multivoltine life cycle (three or four generations a year; Hayashi & Nakazawa, 1988; Kashin et al., 2009), and overwinters in the egg stage (Goto et al., 2000; Hayashi, 1986; Iimura, 1992; Kashin et al., 2009; Nagasawa & Higuchi, 2012). *T*. *caelestialium* has similar host plant preferences and a similar life history to *S. rubrovittatus* (Nagasawa & Higuchi, 2012; Okuyama, 1974; Okuyama & Inouye, 1975), except for its preference for ovipositioning in the tight gap between the stems and leaves of host plants (Nagasawa et al., 2012). Both species are important pests of cultivated rice, and first‐ or second‐generation adults invade paddy fields and feed on rice ears in August, which is the heading period, causing pecky rice damage (Ono et al., 2007). Crop damage by these species has become a serious problem in the Tohoku region of Japan since around 2000. In the first half of the 2000s, *T*. *caelestialium* appeared as a major pest species in the prefectures on the Sea of Japan side, including Akita, and *S. rubrovittatus* appeared as a major pest species in the prefectures on the Pacific side, including Iwate. Since around 2007, *S. rubrovittatus* became the main pest species also in Akita (Tabuchi et al., 2015).

### 2.2 Development experiments

**FIGURE 1:**
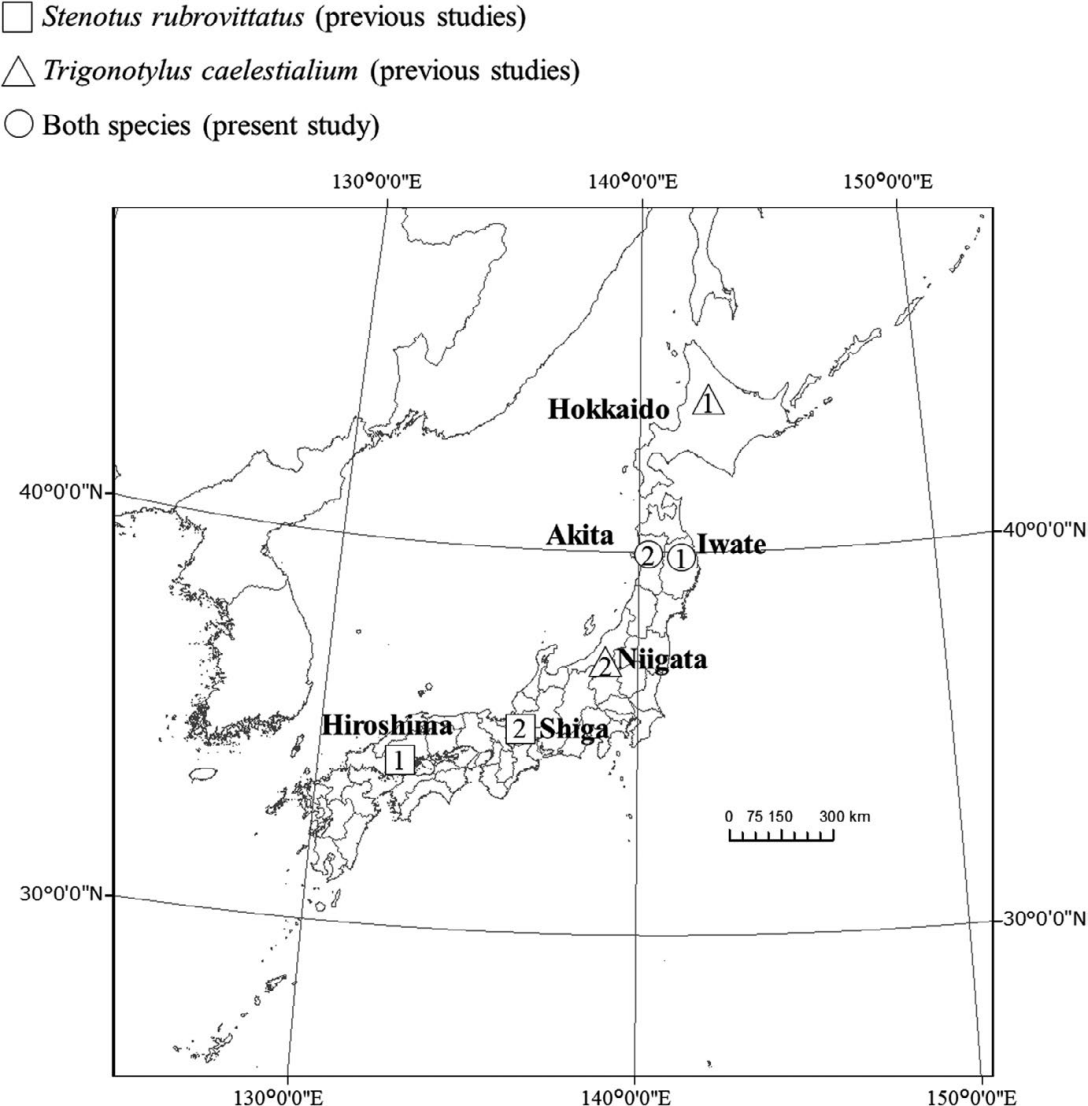
Sites where *T*. *caelestialium* and *S. rubrovittatus* were collected. ○ Both species collected within this study in (1) Iwate Prefecture in 2016 and (2) Akita Prefecture in 2017. ∆ *T*. *caelestialium* collected by (1) Okuyama and Inouye (1975) and (2) Takahashi and Higuchi (2001). □ *S. rubrovittatus* collected by (1) Hayashi (1991) and (2) Shigehisa (2004). Coastlines and boundaries were obtained from the Database of Global Administrative Areas (https://gadm.org/)

We collected *S. rubrovittatus* and *T*. *caelestialium* in Morioka city, Iwate Prefecture (39°45′11.82″N 141°8′9.20″E), in October 2016, and in Akita city, Akita Prefecture (39°36′55.3″N 140°11′15.7″E), in June 2017 (Figure 1). Both species were raised using wheat seedlings as food for 1 or 2 months about at 27°C in the laboratory according to the methods of Higuchi and Takahashi (2000) and Nagasawa and Higuchi (2010), in transparent acrylamide cages (34 × 25 × 34 cm^3^, Fujiwara Manufacturing Co., Ltd.). To compare the rates of development between the two populations under experimental conditions where only the temperature differed between trials, we reared the bugs at 30, 27, 23.5, 20, and 17.5°C under 16L8D photoperiod. We selected the temperature range for the following two reasons. First, the four previous studies we used in this study set a similar range (Appendix S1). Second, 30 and 17.5°C correspond roughly to the maximum and minimum daily temperatures, respectively in Iwate and Akita Prefectures during active seasons of the two mirid bugs (from June to September) in 2013. We set the humidity at 60% in our experiments. We checked the temperature and humidity a few times a day to keep them at plus or minus 0.5°C and plus or minus 10%, respectively. Eggs were placed in a Petri dish within 24 h after oviposition, and incubated according to Nagasawa and Higuchi (2010). We recorded the number of days from egg collection day to hatching as the “egg stage.” We introduced hatched nymphs individually into insect breeding boxes (7.2 cm × 7.2 cm × 10 cm, SPL Life Sciences). After hatching, we gave six wheat seedlings (1 week after sowing) wrapped in wet cotton to each as food. The food was replaced once every 2–6 days, and the number of days from hatching to emergence was recorded as the “nymphal stage.” After emergence, only the adult females continued to be reared in the breeding boxes, and one male adult of the same population was introduced into each female box for copulation. We provided 6‐week‐old wheat seedlings as food and 2‐day‐old seedlings as oviposition substrate. The seedlings were replaced every day, and the presence of eggs was confirmed by dissection of the seedlings. We recorded the number of days from the date of emergence until the day when the first oviposition was confirmed as the “preoviposition stage.” We checked all bugs from hatching to the day when the first oviposition once a day. The rates of development at each temperature were calculated as the reciprocals of the developmental days required for each stage. The developmental zero points and the effective accumulated temperatures (EATs) of each stage were calculated from the reduced major axis regression (Ikemoto & Takai, 2001).

To test any intraspecific variation in body size between the populations, we measured the thorax width of the adults as an index of body size. The reason why we used the thorax size as an index of body size is because it is not affected by short‐term food quantity very much, and many previous studies have used thorax size as an index of body size of insects (Chown & Gaston, 2010). We measured females and males reared at 30 and 27°C, at which many adults were obtained. The widths were measured under a stereoscopic microscope with an objective micrometer.

### 2.3 Literature survey

Data on the average development rates of the egg, nymph, and preoviposition stages of *S. rubrovittatus* at different temperatures in Shiga and Hiroshima Prefectures were obtained from Shigehisa (2004) and Hayashi (1991), respectively. Data on those of *T*. *caelestialium* at different temperatures in Hokkaido and Niigata Prefectures were obtained from Okuyama and Inouye (1975) and Takahashi and Higuchi (2001), respectively. We selected these four studies because they have examined the developmental rates of most of the egg, nymphal, and preoviposition stages of the two mirid bug species, with sufficient sample sizes (Appendix S1). On the other hand, we could not access data on the rate of development of each individual, so we used the mean values in the statistical analyses.

### 2.4 Statistical analyses

To test whether the rate of development of each stage differs between the Iwate and Akita populations of each species, we used general linear mixed effect models with the rate of development of each individual as the dependent variable. The model for the egg and nymphal stages includes temperature, population (Iwate vs. Akita), their interaction, and sex of the individuals as fixed factors with each level of each of the three experimental treatments as a random factor. The model for preoviposition stage includes temperature, population (Iwate vs. Akita), and their interaction as fixed factors with each level of each of the two experimental treatments as a random factor.

Then, we compared the rates among the Tohoku (Iwate and Akita) and two other Prefectures of each species at different latitudes using data in the previous studies (Figure 1; Appendix S1). We used eight candidate general linear models explaining the rate of development of each stage of each species (Table 1). Each model aims to identify one of the possible scenarios between development and temperature across a range of temperatures that differed among studies. The aim was to select the best model for each stage to identify which parameters were responsible per case. Due to the very low variance of the mean development rate in the four previous studies and our experiments, only the mean values were used in the following analysis. Model 1 assumes that the rate differed among the three populations was affected by temperature, explaining that these two predictor variables affected the rate independently. Model 2, including the interaction term of the two predictor variables, explains that the effect of temperature on the rate differed among the populations. Models 3 explains that the rate differed between one population and the other two (Hiroshima vs. Tohoku‐Shiga for *S. rubrovittatus*, Hokkaido vs. Tohoku‐Niigata for *T*. *caselestialium*) and was also affected by temperature. Model 4, adding the interaction term to Model 3, explains that the effect of temperature on the rate differed between one population and the other two. Model 5 explains that the rate differed between one population and the other two (Tohoku vs. Shiga‐Hiroshima for *S. rubrovittatus*, Niigata vs. Tohoku‐Hokkaido for *T*. *caselestialium*) and was also affected by temperature. Model 6, including the interaction term to Model 5, explains that the effect of temperature on the rate differed between one population and the other two. Model 7 explains that the rate was affected only by temperature. Model 8, with only the intercept term, is the null model. Model selection was performed using Akaike's Information Criteria corrected for small sample size (AIC_c_; Burnham & Anderson, 2002). A lower AIC_c_ value is considered to indicate a better model. AIC_c_ was calculated for each model and models with ΔAIC_c_ (the difference between the AIC_c_s of a focal model and that of the model having the lowest AIC_c_) < 2 were chosen as optimal models. We selected the model with the fewest predictor variables among the optimal models. To test whether the rate of development of each stage differs between the two species in the Tohoku region, we used GLMs with the development rate as the dependent variable, and temperature, species (*S. rubrovittatus* vs. *T. caelestialium*), and their interaction as the predictor variables.

**TABLE 1:** Models of the development rates of *S. rubrovittatus* and *T*. *caelestialium* in three latitudinally different populations of each species

No.	Models
*S. rubrovittatus*	
1	Three populations + temperature
2	Three populations + temperature + three populations × temperature
3	Populations (Tohoku‐Shiga vs. Hiroshima) + temperature
4	Populations (Tohoku‐Shiga vs. Hiroshima) + temperature +populations (Tohoku‐Shiga vs. Hiroshima) × temperature
5	Populations (Tohoku vs. Shiga‐Hiroshima) + temperature
6	Populations (Tohoku vs. Shiga‐Hiroshima) + temperature +populations (Tohoku vs. Shiga‐Hiroshima) × temperature
7	Temperature
8	Intercept
*T. caelestialium*	
1	Three populations + temperature
2	Three populations + temperature + three populations × temperature
3	Populations (Hokkaido vs. Touhoku‐Niigata) + temperature
4	Populations (Hokkaido vs. Tohoku‐Niigata) + temperature +populations (Hokkaido vs. Tohoku‐Niigata) × temperature
5	Populations (Hokkaido‐Tohoku vs. Niigata) + temperature
6	Populations(Hokkaido‐Tohoku vs. Niigata) + temperature + populations(Hokkaido‐Tohoku vs. Niigata) × temperature
7	Temperature
8	Intercept

To test whether the morphology data differ between the Iwate and Akita populations of each species, we used GLMs with the thorax width as the dependent variable, and sex, population (Iwate vs. Akita), and their interaction as the predictor variables.

All analyses were carried out in R v. 3. 3. 1 software (R Development Core Team, 2016).

### 2.5 Estimation of date of theoretical adult emergence and the number of generations per year

Using the method of Osawa et al. (2018b), we estimated the dates of adult emergence of the first generation and the theoretical number of annual generations of *S. rubrovittatus* and *T*. *caelestialium* in Tohoku (Iwate and Akita) in 2013 using the daily mean temperature mesh data of the National Institute for Agro‐Environmental Sciences and our data on the two species’ EAT and the developmental zero points. Each 5‐km grid cell corresponding to the records of *S. rubrovittatus* and *T*. *caelestialium* occurrence has 25 daily temperature data units (Osawa et al., 2018b). The effective temperature was estimated by the triangle method (Sakagami & Korenaga, 1981) setting 1 April as a start date (Murakami et al., 2012; Yokota & Suzuki, 2008). First, we estimated the theoretical egg hatching date from 1 April using the EAT and the developmental zero point of the egg stage of each species obtained from our rearing experiments (see Section 3). Second, we estimated the durations of the nymphal stage of each species from the date when eggs hatched using the EAT and the developmental zero point of the nymphal stage. Finally, we estimated the reproductive maturation date (the end of the preoviposition stage) from the date when the nymphal stage ended using the EAT and the developmental zero point of the preoviposition stage. This series of calculations provided an estimate of the generation time for each species’ overwintered generation. The first and subsequent generation cycles of each species were estimated using the same procedure from the date of reproductive maturation (i.e., start of the oviposition stage) of the proximate generation. Thus, we calculated the span of dates for each development stage and the theoretical generation number in each 1‐km grid cell for each species. We ignored the shift from nondiapause to diapause egg production induced by short‐day conditions (Okuyama, 1982; Shigehisa, 2008). If a generation did not reach the egg‐laying stage within a calendar year, it was not counted. In this study we used temperature data of 2013 for the estimation, because *S. rubrovittatus* was recorded in a low population density in the prefectures on the western side in Tohoku region including Akita Prefecture around 2000, gradually increased from 2003 to 2012, and both two mirid bug species have been recorded throughout the Tohoku region since around 2013 (Osawa et al., 2018b; Tabuchi et al., 2015). We focused on the first‐generation adults because the emergence day of this generation overlaps the heading stage of rice, when pecky rice damage occurs (Ono et al., 2007).

## 3 RESULTS

### 3.1 Comparison of development rates at two spatial scales

**FIGURE 2:**
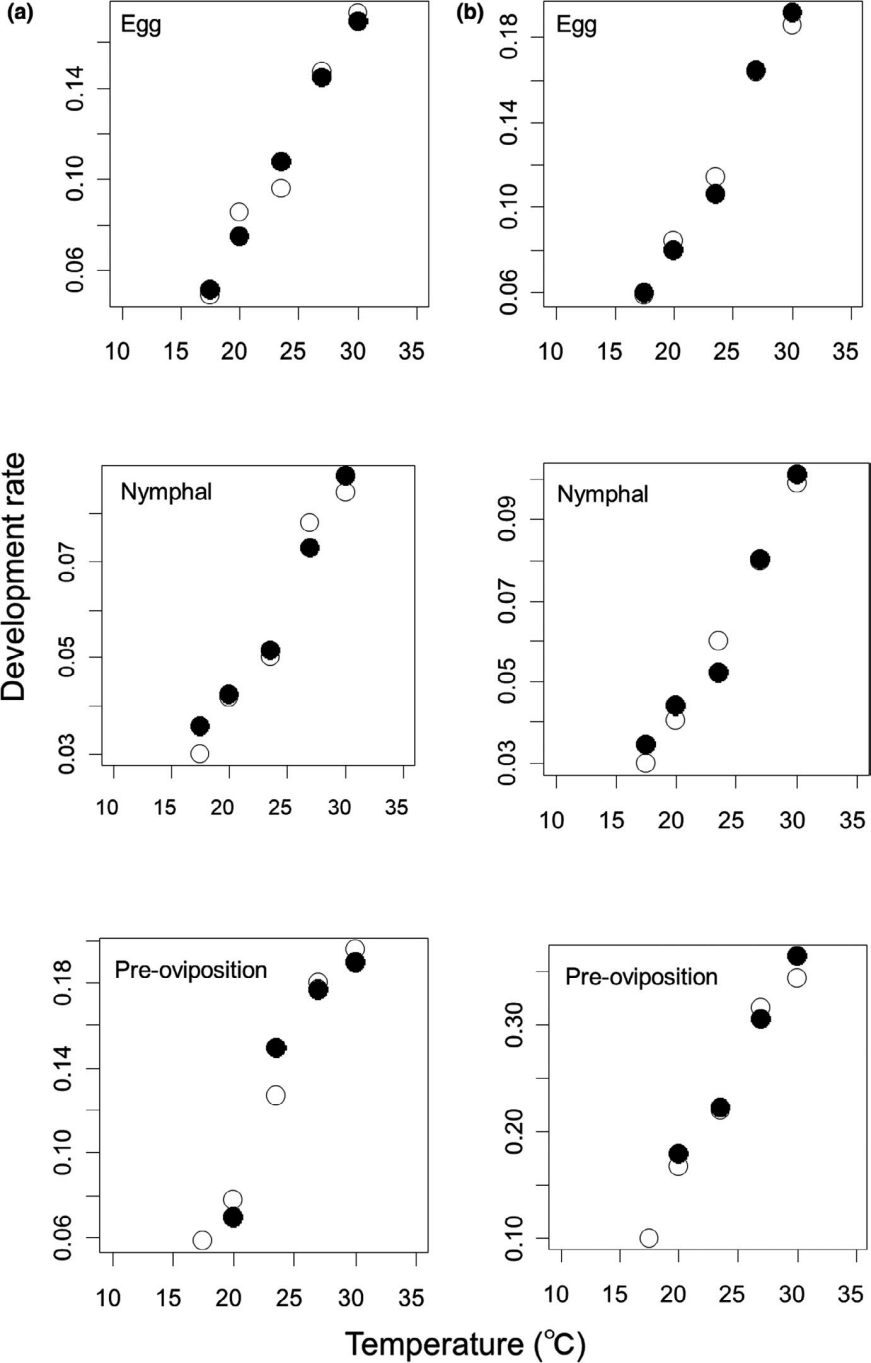
Relationships between temperature and development rate of (a) *S. rubrovittatus* and (b) *T*. *caelestialium* in Iwate (○) and Akita (●) populations at each developmental stage

**FIGURE 3:**
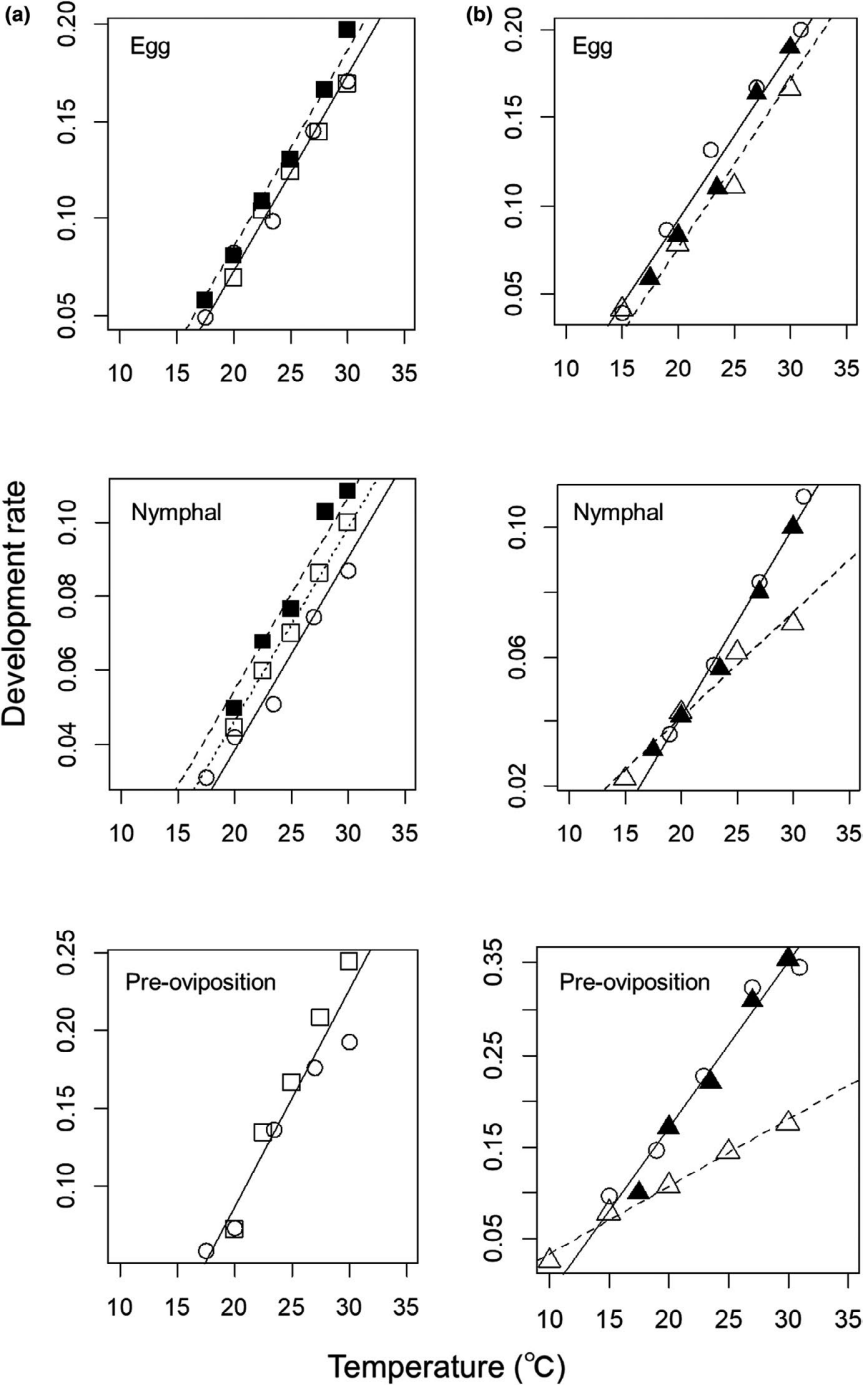
Relationships between temperature and development rate of (a) *S. rubrovittatus* in Tohoku region (○), Shiga (□), and Hiroshima (■) Prefectures and (b) *T*. *caelestialium* in Tohoku region (○), Niigata (▲), and Hokkaido (∆) Prefectures at each developmental stage. Each line indicates the relationship between temperature and development rate in each population

The rate of development at each stage was not significantly different between the Iwate and Akita populations of *S. rubrovittatus* (Table 2; Figure 2a) or *T*. *caelestialium* (Table 3; Figure 2b). In addition, the effect of sex was also not significant (Tables 2 and 3). We combined both populations into the “Tohoku population” for each species. Model selection among the three populations (Hiroshima, Shiga, and Tohoku populations) of *S. rubrovittatus* at different latitudes identified Model 3 (the rate of development differs between the Hiroshima and the other two populations (Shiga and Tohoku populations) and is affected by temperature) as the best for the egg stage; Model 1 (it differs among the three populations and is affected by temperature) as the best for the nymphal stage; and model 7 (it is related only to temperature) as the best for the preoviposition stage (Table 4). For *S. rubrovittatus*, the rate of development increased with temperature and was higher in Hiroshima than in the other two populations in the egg stage (Figure 3a). In the nymphal stage, the rate of development increased with temperature and was the highest in southernmost population (Hiroshima) and the lowest in northernmost population (Tohoku; Figure 3a). In the preovipositon stage, the rate of development increased with temperature but did not differ among three populations (Figure 3a). Model selection among the three populations of *T*. *caelestialium* at different latitudes identified Model 3 (the rate differs between the Hokkaido and the other two populations (Tohoku and Niigata populations) and is affected by temperature) as the best for the egg stage; and Model 4 (the effect of temperature on the rate differs between Hokkaido and the other two populations (Tohoku and Niigata populations)) as the best for both the nymphal and preoviposition development stages (Table 4). For *T*. *caelestialium*, the rate of development increased with temperature and was lower in Hokkaido than in the other two populations in the egg stage (Figure 3b). In the nymphal and preoviposition stages, the rate of development was lower in the Hokkaido than in the other two populations and the difference was greater at higher temperatures (Figure 3b).

**TABLE 2:** Effects of each parameter on the development rates of *S. rubrovittatus* in Iwate and Akita

Stage	Parameter	Numerator *df*	Denominator *df*	*F*	*p*
Egg	Temperature	1	15	713.2863	<.001
	Population	1	15	0.0079	.9304
	Temperature × population	1	15	0.0160	.9010
	Sex	1	15	0.0396	.8450
Nymph	Temperature	1	15	177.7902	<.001
	Population	1	15	0.0003	.9864
	Temperature × population	1	15	0.0002	.9893
	Sex	1	15	2.9207	.1081
Preoviposition	Temperature	1	5	33.6871	.0021
	Population	1	5	0.0011	.9748
	Temperature × population	1	5	0.0002	.9900

**TABLE 3:** Effects of each parameter on the development rates of *T*. *caelestialium* in Iwate and Akita

Stage	Parameter	Numerator *df*	Denominator *df*	*F*	*p*
Egg	Temperature	1	14	846.8975	<.001
	Population	1	14	2.2722	.1539
	Temperature × population	1	14	2.3549	.1472
	Sex	1	14	0.0128	.9115
Nymph	Temperature	1	14	547.4894	<.001
	Population	1	14	0.0160	.9011
	Temperature × population	1	14	0.0097	.9230
	Sex	1	14	1.2371	.2848
Preoviposition	Temperature	1	5	77.1744	<.001
	Population	1	5	0.0151	.9070
	Temperature × population	1	5	0.0212	.8899

**TABLE 4:** Eight candidate models explaining the development rate in each stage of *S. rubrovittatus* and *T*. *caelestialium* and their information‐theoretic statistics

Model	Variables included in the model			Egg		Nymph		Preoviposition^a^	
				AICc	ΔAICc^b^	AICc	ΔAICc^b^	AICc	ΔAICc^b^
*S. rubrovittatus*									
1	Temperature	Population (Tohoku, Shiga, vs. Hiroshima)		−106.5	5.47	−109.9	0.00	−42.5	1.76
2	Temperature	Population (Tohoku, Shiga, vs. Hiroshima)	Their interaction	−99.9	11.99	−107.2	2.70	−42.3	1.96
3	Temperature	Population (Tohoku‐Shiga vs. Hiroshima)		−110.8	1.16	−105.8	4.05	−	−
4	Temperature	Population (Tohoku‐Shiga vs. Hiroshima)	Their interaction	−111.9	0.00	−103.2	6.71	−	−
5	Temperature	Population (Tohoku vs. Shiga‐Hiroshima)		−99.5	12.38	−104.5	5.33	−	−
6	Temperature	Population (Tohoku vs. Shiga‐Hiroshima)	Their interaction	−95.8	16.17	−104.1	5.78	−	−
7	Temperature			−100.9	11.04	−96.1	13.81	−44.29	0.00
8	Intercept only			−50.3	61.58	−65.7	44.21	−22.32	21.96
*T. caelestialium*									
1	Temperature	Population (Hokkaido, Tohoku, vs. Niigata)		−79.66	2.98	−74.36	21.83	−42.02	28.06
2	Temperature	Population (Hokkaido, Tohoku, vs. Niigata)	Their interaction	−74.78	7.86	−81.58	14.62	−62.46	7.63
3	Temperature	Population (Hokkaido vs. Touhoku‐Niigata)		−81.38	1.26	−79.51	16.69	−46.65	23.44
4	Temperature	Population (Hokkaido vs. Touhoku‐Niigata)	Their interaction	−82.64	0.00	−96.19	0.00	−70.09	0.00
5	Temperature	Population (Hokkaido‐Tohoku vs. Niigata)		−80.43	2.21	−77.53	18.66	−37.67	32.41
6	Temperature	Population (Hokkaido‐Tohoku vs. Niigata)	Their interaction	−75.82	6.82	−76.14	20.05	−33.77	36.31
7	Temperature			−77.80	4.84	−80.12	16.07	−38.99	31.09
8	Intercept only			37.78	120.42	−52.99	43.20	−21.29	48.80

^a^ No data was available from Hayashi (1991).

^b^ ΔAICc is the difference between the AICcs of a focal model and that of the model having the lowest AICc.

**FIGURE 4:**
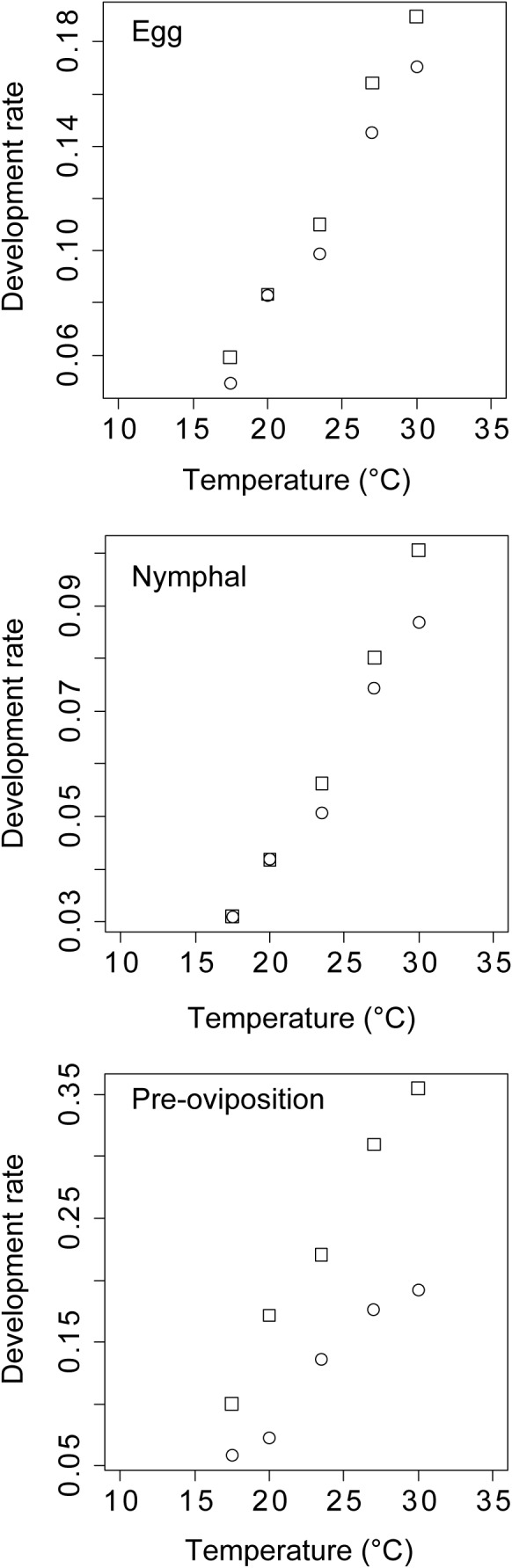
Relationships between temperature and development rate of *S. rubrovittatus* (○) and *T*. *caelestialium* (□) in Tohoku populations

There was no significant difference in the rates of development between species in Tohoku at the egg and nymphal stages, but the species × temperature interaction was significant at the preoviposition stage (Table 5; Figure 4). This result indicates that the effect of temperature on the rate of development differs between the two species only at the preoviposition stage. The developmental zero points (i.e., the lower developmental threshold) of *S. rubrovittatus* were 12.4°C in the egg stage, 10.7°C in the nymphal stage, and 13.2°C in the preoviposition stage, and those of *T*. *caelestialium* were 12.0, 11.9, and 12.7, respectively. The EATs of *S. rubrovittatus* were 102.0 degree‐days (°C) in the egg stage, 226.3 degree‐days in the nymphal stage, and 81.8 degree‐days in the preoviposition stage, and those of *T*. *caelestialium* were 95.9, 190.5, and 47.0 degree‐days, respectively.

**TABLE 5:** Effects of each parameter on the development rates of *S. rubrovittatus* and *T*. *caelestialium* in Tohoku

Stage	Parameter	*Df*	Sum Sq	*F*	*Pr(>F)*
Egg	Species	1	0.0000	0.4471	0.5286
	Temperature	1	0.0118	222.2840	<0.001
	Species × temperature	1	0.0001	1.3827	0.2842
	Residuals	6	0.0003		
Nymphal	Species	1	0.0000	2.3140	0.1790
	Temperature	1	0.0032	215.5026	<0.001
	Species × temperature	1	0.0001	3.7696	0.1002
	Residuals	6	0.0001		
Preoviposition	Species	1	0.0009	5.7406	0.0536
	Temperature	1	0.0418	281.3929	<0.001
	Species × temperature	1	0.0038	25.3141	0.0024
	Residuals	6	0.0009		

### 3.2 Body size comparison

**FIGURE 5:**
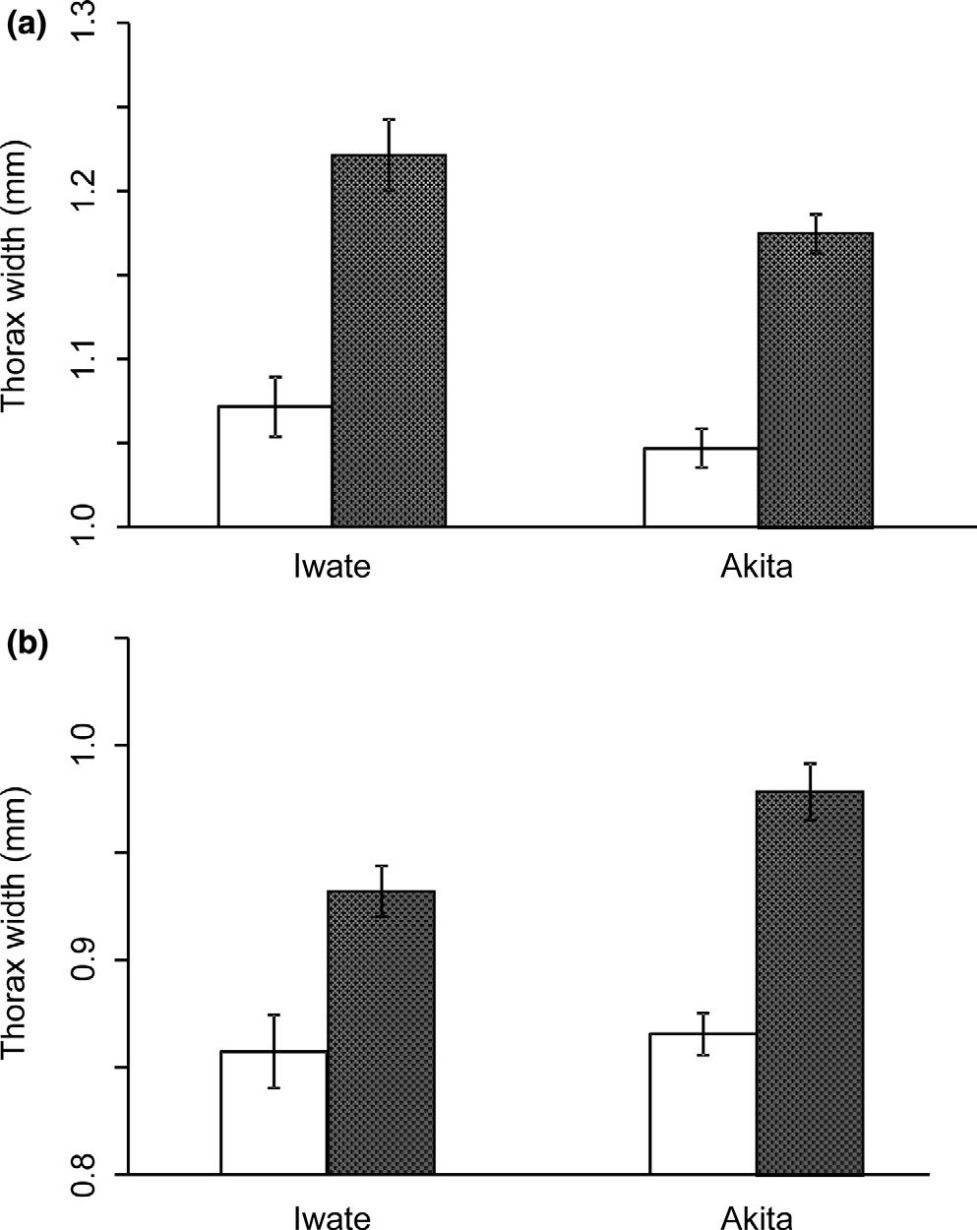
Thorax width (mean ± SE) of male (□) and female (■) (a) *S. rubrovittatus* and (b) *T*. *caelestialium* in Iwate and Akita populations

We measured the thorax widths of 82 *S. rubrovittatus* and 94 *T*. *caelestialium* females and males reared in the 30 and 27°C treatments. The thorax width of *S. rubrovittatus* was significantly larger in the females than in the males, and in Iwate than in Akita, but the interaction effect between population and sex was not significant (Table 6; Figure 5). In *T*. *caelestialium*, it was also significantly larger in the females, and was significantly larger in Akita than in Iwate, but the interaction effect between population and sex was not significant (Table 7; Figure 5).

**TABLE 6:** Effects of each parameter on the thorax width of *S. rubrovittatus*

Parameter	*Df*	Sum Sq	*F*	*Pr(>F)*
Population	1	0.0210	4.9147	0.0294
Sex	1	0.3270	76.1879	<0.001
Population × sex	1	0.0020	0.4416	0.5082
Residuals	81	0.3480		

**TABLE 7:** Effects of each parameter on the thorax width of *T. caelestialium*

Parameter	*Df*	Sum Sq	*F*	*Pr(>F)*
Population	1	0.0170	4.4743	0.0371
Sex	1	0.2000	52.3055	<0.001
Population × sex	1	0.0090	2.2384	0.1380
Residuals	93	0.3550		

### 3.3 Estimation of date of theoretical adult emergence and generations per year

**FIGURE 6:**
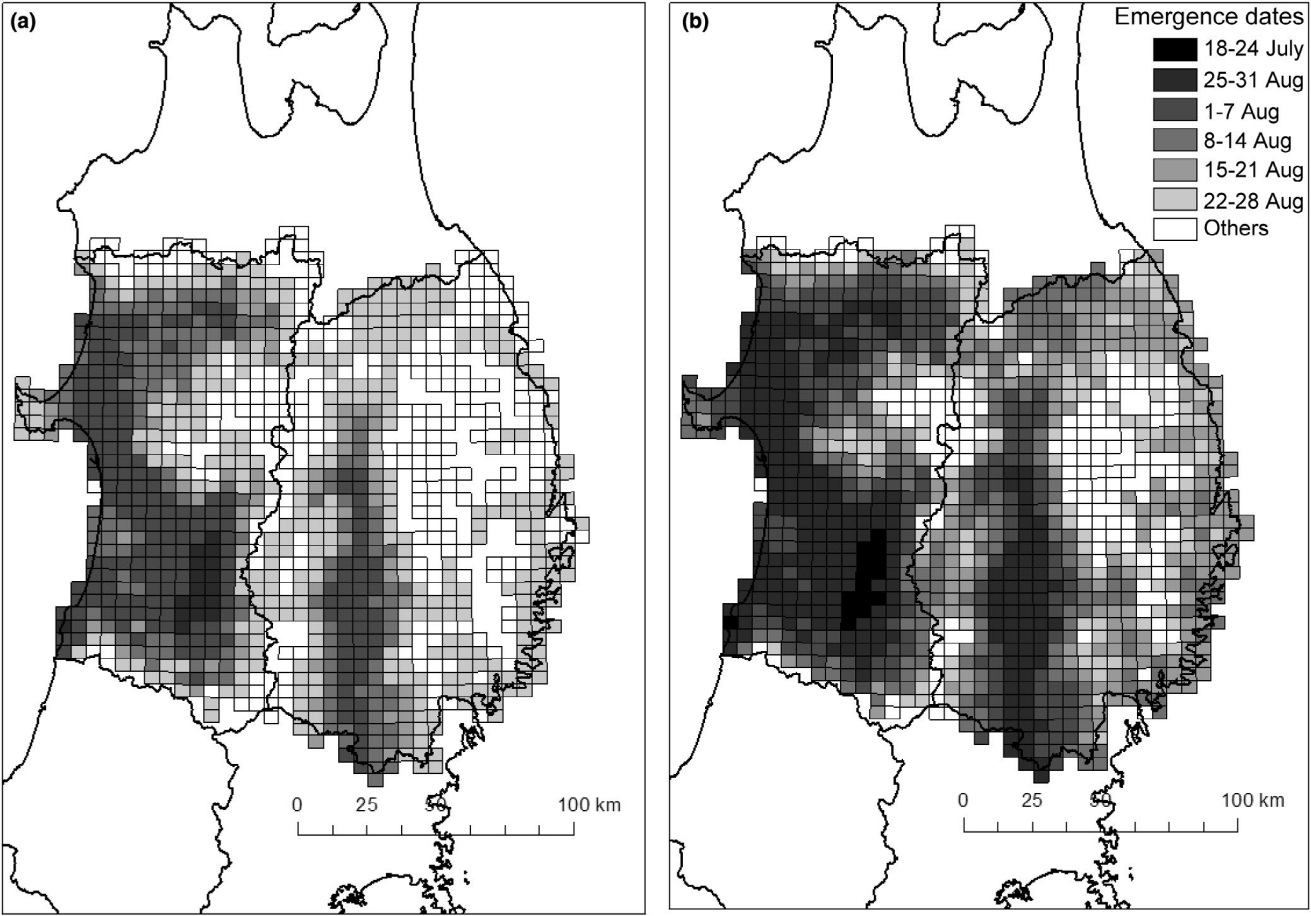
Average theoretical emergence dates of first generation of (a) *S. rubrovittatus* and (b) *T*. *caelestialium* in 2013 based on their EAT and developmental zero points. Grid cells having white color (Others) could not be calculated the emergence dates of first generation due to low temperature

**FIGURE 7:**
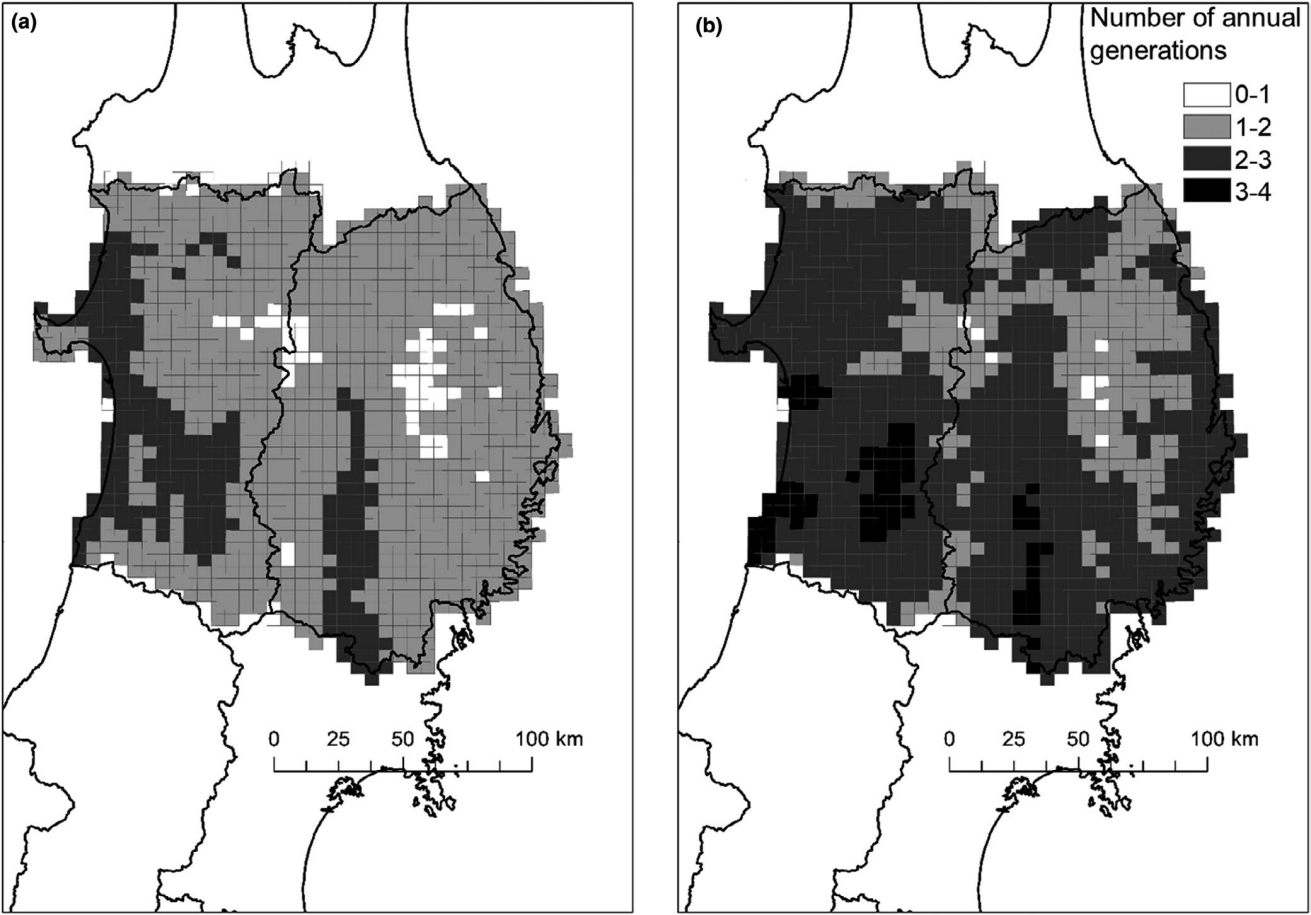
Average theoretical number of annual generations of (a) *S. rubrovittatus* and (b) *T*. *caelestialium* in 2013 based on their EAT and developmental zero points

The date of first emergence of *S. rubrovittatus* adults in 2013 was estimated to be 29 July, and the median was 19 August (Figure 6a). Those of *T*. *caelestialium* were 22 July and 11 August, respectively (Figure 6b). In *S. rubrovittatus*, 84.5% of the 5‐km grid cells in Iwate and Akita showed successful completion of the second generation, and 27.2% of the cells (some inland cells in Iwate and some coastal cells in Akita) showed successful completion of the third generation as well (Figure 7a). In *T*. *caelestialium*, 39.3%, 54.5%, and 2.7% of the cells successfully completed the second, third fourth generations, respectively (Figure 7b).

## 4 DISCUSSION

Our laboratory rearing experiments revealed no difference in the relationship between temperature and the rate of development in each developmental stage of the two mirid bug species from the same latitude in northern Japan. On the other hand, comparison of data on the development rates in Tohoku region and those of previous studies in other areas revealed that the relationship between temperature and the rate of development showed geographical variations between populations of both species at different latitudes.

The rate of development of *S. rubrovittatus* was higher in Hiroshima, the southernmost of the three populations, than in Shiga and Tohoku in the egg stage, and was the highest in southernmost population (Hiroshima) and the lowest in northernmost population in the nymphal stage (Tohoku; Figure 3a). For *T*. *caelestialium*, the rate of development was lower in Hokkaido, northernmost of the three populations, than in Tohoku and Niigata in the egg stage, and the difference was greater at higher temperatures in the nymphal and preoviposition stages (Figure 3b). The adaptive significance of the geographic intraspecific variation in development rates has not been adequately validated in many insect species (Kipyatkov & Lopatina, 2010). The relationship between latitude and growth rate of insects is known to be diverse (Blackenhorn & Demont, 2004). Positive relationship between latitude and growth rate has been reported in univoltine species with less time available for growth in higher latitudes (e.g., Kojima et al., 2020), and conversely, multivoltine species in lower latitudes is reported to have more generations per year, resulting in negative relationship between latitude and growth rate (e.g., Stoks et al., 2012). Higher development rates in lower‐latitude populations of *S. rubrovittatus* and *T*. *caelestialium*, having multivoltine life cycles (see Section 2), may increase the number of generations. On the other hand, the higher‐latitude populations are likely to be hard to increase the number of generations due to temperature constraint.

In this analysis, it cannot be ignored that the diets used in the rearing experiments differed among our and the two previous studies (Hayashi, 1991; Okuyama & Inouye, 1975; Appendix S1). The response of insect development to temperature depends on diet (Ayres & Scribner, 1994; Goryshin et al., 1988). The Hiroshima population of *S. rubrovittatus* in Hayashi (1991) was reared on ears of *Lolium multiflorum*, one of the plants most preferred by the mirid bug (Nagasawa & Higuchi, 2012), and this may have caused the high development rate. The Hokkaido population of *T*. *caelestialium* in Okuyama and Inouye (1975) was reared on rice leaves, which are less preferred than some grass and sedge weeds, and this may have caused the lower development rate. On the other hand, we found that the rate of development of *S. rubrovittatus* in Tohoku revealed by our experiments was lower than that in Shiga population at nymphal stage (Figure 3a). The data in Shiga population was obtained from Shigehisa (2004) using the same diet in rearing experiments as ours (Appendix S1). We also revealed that the difference in development rates of the two species was not observed between populations of the same latitude in the Tohoku region by our rearing experiments. In addition, we showed that the relationship between temperature and the rate of development of *T*. *caelestialium* in the Tohoku region did not differ from that of Niigata Prefecture obtained from Takahashi and Higuchi (2001) using the same diet in rearing experiments as ours. To clarify the extent of geographic variation in temperature‐dependent development among populations of the two mirid bugs more clearly, it is necessary to conduct similar rearing experiments with many populations from wider range of latitudes.

The adult body size of both sexes of *S. rubrovittatus* was larger in Iwate than in Akita and that of *T*. *caelestialium* was larger in Akita than in Iwate (Tables 6 and 7; Figure 5), but there was no difference in the rates of development of each stage between the Iwate and Akita populations. We consider the two populations of the two species different genetically, as variations in body size were observed in those reared under the same experimental conditions. One reason for their genetic difference despite the short distance of about 85 km between the collection sites is the presence of the Ou Mountains, with peaks of up to 2000 m in elevation between the prefectures. Population dynamics may differ between the two populations in both species, as it is known that female fertility and survival rate in insects increase with body size (Blanckenhorn, 2000; Honěk, 1993; Lighton et al., 1994; Parker & Simmons, 1994; Rivero & West, 2002). The body size also differed between the sexes: the females were significantly larger in both species (Tables 6 and 7; Figure 5), but the population × sex interaction was not significant in either species (Tables 6 and 7). These results are consistent with the report that female *T*. *caelestialium* have longer forewing length (Higuchi & Takahashi, 2000; Nagasawa & Higuchi, 2012). It may affect the reproductive success of the two mirid bug species.

We mapped the timing of adult emergence of the first generation and the potential number of generations per year only within Iwate and Akita Prefectures using the daily mean temperature in 2013 and our data on the two species’ development in an EAT model, because our results showed no difference in the temperature dependence of development between populations at the same latitude, but showed geographic variation among populations at different latitudes. The estimation of adult emergence date of the first generation showed about 8 days’ difference between species in both the first day and the median day. Thus, *T*. *caelestialium* adults emerged a week earlier than *S. rubrovittatus* (Figure 6), suggesting that monitoring should be established earlier. In both Iwate and Akita Prefectures, the adult emergence dates differed locally even at the same latitude (Figure 6). This would make the migration pattern of the two species to paddy fields more complex. Future research is needed to determine how the variation in the timing of first adult emergence between and within species affects the timing of movement of these two species into paddy fields and the resulting pecky rice damage level. The estimates suggest that *T*. *caelestialium* can successfully reproduce four generations per year, one generation more than *S. rubrovittatus* in some areas (Figure 7). In Akita, *T*. *caelestialium* was the main species in the early 2000s (Tabuchi et al., 2015). Its rapid increase to major pest status in Akita may be related to its estimated ability to reproduce one more generation. One of the reasons why the estimated number of generations of *T*. *caelestialium* differs by as much as three within a small spatial scale (within 50 km; Figure 7) may be due to the temperature difference caused by the large difference in altitude. For example, the Ou Mountains, with peaks of up to 2000 m in elevation, lie between Akita and Iwate Prefectures. Simultaneous mowing of fallow fields and footpaths, the bugs’ main habitats, is recommended for the control of the flightless nymphs (Watanabe & Higuchi, 2006). The large differences among the estimated dates of adult emergence both within and between species at the 5‐km scale suggest that the optimum timing of such management also differs locally. In addition, there are many places where these two species co‐occur. Our estimates indicated that in the same place, the time of occurrence is significantly different between the two species, suggesting that it will be necessary to manage their main habitats more frequently than before.

Although there was no difference in the rates of development between populations at the same latitude, the results support our hypothesis that the relationship between temperature and rate of development differs between populations at different latitudes, being higher in the southern populations. However, the extent of the difference depends on species and development stage. These results suggest that differences in the relationship between temperature and rate of development among insect populations are not determined at a specific developmental stage, and investigating only specific stages may reduce the accuracy of results. Therefore, it is important to investigate development rates throughout the entire life cycle.

Our estimates of the dates of adult emergence and the numbers of annual generations revealed that the timing of emergence differs between the two mirid bug species by about a week, and differs greatly even within a species locally at the same latitude. By combining the rates of development of each species in the Tohoku region, northern Japan with temperature forecasts, it may be possible to predict when and where the first generation adults of each species emerge each year. This technique could be used for developing more efficient methods of managing pecky rice bugs in integrated pest management. In addition, global warming is known to contribute to the increase in the density and distribution of these two species (Kiritani, 2007; Osawa et al., 2018a). By predicting where the number of generations of these two species will increase in the future as global warming progresses, it will be possible to identify where we should manage priority.

## CONFLICT OF INTEREST

The authors declare that they have no conflict of interest.

## AUTHOR CONTRIBUTION

**Kazuhisa Yamasaki:** Data curation (equal); Formal analysis (equal); Investigation (lead); Writing‐original draft (lead). **Ken Tabuchi:** Data curation (equal); Funding acquisition (equal); Methodology (equal); Writing‐review & editing (equal). **Akihiko Takahashi:** Investigation (supporting); Methodology (lead); Resources (equal); Writing‐review & editing (supporting). **Takeshi Osawa:** Data curation (equal); Funding acquisition (equal); Investigation (equal); Methodology (lead); Project administration (equal); Resources (equal); Writing‐review & editing (equal). **Akira Yoshioka:** Funding acquisition (equal); Methodology (equal); Writing‐review & editing (equal). **Yasushi Ishigooka:** Formal analysis (lead); Methodology (equal); Resources (equal); Writing‐review & editing (supporting). **Shigeto Sudo:** Formal analysis (lead); Resources (equal); Writing‐review & editing (supporting). **Mayura B. Takada:** Conceptualization (lead); Data curation (lead); Formal analysis (lead); Funding acquisition (lead); Investigation (equal); Methodology (lead); Project administration (lead); Resources (lead); Supervision (lead); Visualization (equal); Writing‐original draft (lead); Writing‐review & editing (lead).

## Supporting information

Appendix S1

## Data Availability

The data that support the findings of this study are archived in the Dryad system https://doi.org/10.5061/dryad.ttdz08m0d.
