# Supplementary material for: Intraspecific variations in life history traits of two pecky rice bug species from Japan: Mapping emergence dates and number of annual generations
Source: Ecol Evol. 2021 Nov 12;11(23):16936–50. doi: 10.1002/ece3.8329 (PMC8668747; doi:10.1002/ece3.8329)
Supplement: Supplementary file 1 — Appendix S1 [file ECE3-11-16936-s001.docx]

**Appendix S1** Information on our experiments and the four previous studies used in this study

|  |  |  |  |  |  |
| --- | --- | --- | --- | --- | --- |
| Species | Prefecture | Temperature of the rearing experiment (°C) | Sample size of each treatment | Diet in rearing | Source |
| *Stenotus rubrovittatus* | Shiga | 20, 22.5, 25, 27.5, 30 | 11 - 133 | wheat | Shigehisa (2004) |
|  | Hiroshima | 17.5, 20, 22.5, 25, 28, 30 | 13 -109 | *Lolium multiflorum* | Hayashi and Nakazawa (1988) |
|  | Iwate and Akita | 17.5, 20, 23.5 27, 30 | 2 - 144* | wheat | This study |
|  |  |  |  |  |  |
| *Trigonotylus caelestialium* | Hokkaido | 10, 15, 20, 25.30 | 21- 228 | rice leaves | Okuyama and Inoue (1975) |
|  | Niigata | 15, 19, 23, 27, 31 | 20-130 | wheat | Takahashi and Higuchi (2001) |
|  | Iwate and Akita | 17.5, 20, 23.5 27, 30 | 2 - 25* | wheat | This study |

*Only the pre-oviposition stage at 17.5°C had a low number of samples, probably because the temperature was low for these two species.
